# Choosing Sides: Impact of Prismatic Adaptation on the Lateralization of the Attentional System

**DOI:** 10.3389/fpsyg.2022.909686

**Published:** 2022-06-23

**Authors:** Stephanie Clarke, Nicolas Farron, Sonia Crottaz-Herbette

**Affiliations:** Neuropsychology and Neurorehabilitation Service, Centre Hospitalier Universitaire Vaudois (CHUV), University of Lausanne, Lausanne, Switzerland

**Keywords:** ventral attentional network, spatial representation, prism-induced plasticity, review, fMRI

## Abstract

Seminal studies revealed differences between the effect of adaptation to left- vs. right-deviating prisms (L-PA, R-PA) in normal subjects. Whereas L-PA leads to neglect-like shift in attention, demonstrated in numerous visuo-spatial and cognitive tasks, R-PA has only minor effects in specific aspects of a few tasks. The paucity of R-PA effects in normal subjects contrasts with the striking alleviation of neglect symptoms in patients with right hemispheric lesions. Current evidence from activation studies in normal subjects highlights the contribution of regions involved in visuo-motor control during prism exposure and a reorganization of spatial representations within the ventral attentional network (VAN) after the adaptation. The latter depends on the orientation of prisms used. R-PA leads to enhancement of the ipsilateral visual and auditory space within the left inferior parietal lobule (IPL), switching thus the dominance of VAN from the right to the left hemisphere. L-PA leads to enhancement of the ipsilateral space in right IPL, emphasizing thus the right hemispheric dominance of VAN. Similar reshaping has been demonstrated in patients. We propose here a model, which offers a parsimonious explanation of the effect of L-PA and R-PA both in normal subjects and in patients with hemispheric lesions. The model posits that prismatic adaptation induces instability in the synaptic organization of the visuo-motor system, which spreads to the VAN. The effect is lateralized, depending on the side of prism deviation. Successful pointing with prisms implies reaching into the space contralateral, and not ipsilateral, to the direction of prism deviation. Thus, in the hemisphere contralateral to prism deviation, reach-related neural activity decreases, leading to instability of the synaptic organization, which induces a reshuffling of spatial representations in IPL. Although reshuffled spatial representations in IPL may be functionally relevant, they are most likely less efficient than regular representations and may thus cause partial dysfunction. The former explains, e.g., the alleviation of neglect symptoms after R-PA in patients with right hemispheric lesions, the latter the occurrence of neglect-like symptoms in normal subjects after L-PA. Thus, opting for R- vs. L-PA means choosing the side of major IPL reshuffling, which leads to its partial dysfunction in normal subjects and to recruitment of alternative or enhanced spatial representations in patients with hemispheric lesions.

## Introduction

The pioneering work of Yves Rossetti and his colleagues opened a new chapter in the rehabilitation of cognitive functions (Rossetti et al., 1998). A relatively simple intervention – a brief session of pointing to visual targets while wearing right-deviating prisms – was shown to alleviate signs of left unilateral neglect, as demonstrated in several paper-and-pencil and reading tests. Later studies reported favorable effects on other cognitive deficits, which were associated with right-hemispheric lesions, including postural imbalance, representational neglect, unilateral extinction in dichotic listening tasks, neglect in haptic exploration of space and of objects (Rode et al., 2001, 2006; Frassinetti et al., 2002; McIntosh et al., 2002; Girardi et al., 2004; Jacquin-Courtois et al., 2010; Revol et al., 2020; Hugues et al., 2021). These well documented effects of a brief exposure to right-deviating prisms in patients with right hemispheric lesions contrast with the absence or only minor cognitive effects in normal subjects (Colent et al., 2000; Berberovic and Mattingley, 2003; Striemer et al., 2006; Bultitude et al., 2013; Clarke and Crottaz-Herbette, 2016). When left-deviating prisms are used in the same experimental set-up, normal subjects present signs that are reminiscent of left unilateral neglect. This effect is transient and was shown to impact space representation, such as revealed by tasks of straight-ahead pointing or line bisection, as well as in other cognitive domains, such as attention or hierarchical processing (Schintu et al., 2014, 2017; Michel and Cruz, 2015; Michel, 2016; Striemer et al., 2016; McIntosh et al., 2019). The effect of L-PA in patients with left-hemispheric lesions has been so far investigated only in two studies, of which one reported modulation of neural responses to visual stimuli in large parts of the occipito-temporal cortex (Crottaz-Herbette et al., 2019) and the other improvement of phonemic fluency (Turriziani et al., 2021).

Seminal behavior and imaging studies have addressed the issue of neural mechanisms, which underlie the effect of exposure to right- or left-deviating prisms and several models have been proposed. Putative mechanisms include change in motor behaviors (Striemer and Danckert, 2010), in hemispheric lateralization of the ventral attentional network (VAN) (Clarke and Crottaz-Herbette, 2016) and in interhemispheric inhibition (Boukrina and Chen, 2021) as well as cerebellar contribution to adaptation (Pisella et al., 2005, 2006) and major reorganization of cortical regions involved in visuo-motor recalibration, in realignment of spatial representations and in spatially related cognition (Panico et al., 2020). As pointed out in several reviews, the neural mechanisms by which low-level motor adaptation impacts high-level cognitive functions remain, however, elusive (Rossetti et al., 2015; Panico et al., 2020).

We shall review current evidence from psychophysical, activation and anatomical studies and on this basis propose a model, which offers a parsimonious explanation of the effects of prismatic adaptation. The point we will be making focuses on neural plasticity brought about by prism-induced mismatch between reaching movement and the visual target and its impact on the stability of spatial representations within the attentional network.

## The Attentional Network

Target detection and voluntary orienting of attention were shown to involve different parts of the posterior parietal cortex (Corbetta et al., 2000). Target detection is part of visual orienting of attention to behaviorally relevant exogenous cues. Visual targets, in particular when presented at unexpected locations, yield activation within the right inferior parietal lobule (IPL), which constitutes together with parts of the ventral frontal convexity the VAN. Later studies demonstrated that both the right and left visual space is represented in right but not left IPL, confirming thus the right hemispheric dominance of VAN (Corbetta and Shulman, 2002; Thiel et al., 2004; Shulman et al., 2010; Beume et al., 2015). Functional connectivity investigations demonstrated strong interactions of right IPL with early stage visual areas of either hemisphere (Ruff et al., 2008).

Voluntary orienting of attention, also referred to as endogenous allocation of attention, was shown to depend on more dorsal parts of the posterior parietal cortex, which is referred to as the dorsal attentional network (DAN). Anatomically DAN comprises the superior parietal lobule (SPL) and the intraparietal sulcus (IPS) (Corbetta et al., 2002). Functionally it encodes the contralateral visual space, as shown with paradigms of orienting and re-orienting of spatial attention, saccadic eye movements, visuo-spatial working memory, and conjunction search tasks (Leonards et al., 2000; Corbetta et al., 2002; Müller et al., 2003; Thiel et al., 2004; Silver and Kastner, 2009). The left and right parts of DAN are interconnected and exert mutual inhibitory effect (Koch et al., 2008, 2011; Corbetta and Shulman, 2011).

Historically two influential theories offered explanations for the striking role of the right hemisphere in neglect and hence in attention. Heilman and Valenstein (1979) proposed that each hemisphere mediates attention, whereby the left hemisphere is competent for the contralateral and the right hemisphere for both the contralateral and ipsilateral space. This aspect of hemispheric dominance was subsequently confirmed by activation studies and is highly relevant to our understanding of VAN (Corbetta and Shulman, 2002; Thiel et al., 2004; Shulman et al., 2010; Beume et al., 2015).

Kinsbourne (1977) highlighted another aspect of the attentional theory, the rivalry between hemispheres, positing that each hemisphere directs attention to the contralateral space and their respective activity is kept in balance by interhemispheric inhibition. This aspect of the attentional theory is highly relevant to our understanding of DAN, as subsequently demonstrated in a series of studies in normal subjects and patients with neglect (Corbetta et al., 2005). In the latter, lesions of the right hemisphere lead to hyperexcitability of parietal-motor connections within the left hemisphere, as result of a decrease of right-to-left inhibition; repetitive TMS administered over the left posterior parietal cortex was shown to normalize the overexcitability and to alleviate neglect symptoms (Koch et al., 2008). In normal subjects, a dysfunction of the right posterior parietal cortex, induced temporarily by theta burst TMS, caused a rightward shift in line bisection judgment and increased resting state functional connectivity between the right posterior parietal cortex and the left superior temporal gyrus (Schintu et al., 2021). This interhemispheric effect appears to rely on structural interhemispheric connections, as indicated by its correlation with fractional anisotropy within the posterior callosal pathway.

## Visuo-Motor Coordination, Reaching

The posterior parietal cortex is strongly involved in visuomotor control, including reaching and pointing (Culham et al., 2006). As shown with event-related fMRI paradigms, the medial part of SPL in either hemisphere tends to respond preferentially during reaching (rather than saccade) intentions and is therefore referred to as the parietal reach region (Connolly et al., 2003). This region appears to encode primarily visual information about the target position and not the planned pointing movement (Fernandez-Ruiz et al., 2007). The activation that is associated with pointing involves bilaterally the nearby IPS and is modulated by the position of the target – greater activation for targets in the contra – than ipsilateral visual field – and by the combination of the position of the target and the hand. The latter features stronger activation within IPS when the contralateral hand reaches for a visual target on the same than the opposite side to the hand (Medendorp et al., 2005).

Reach-related spatial representations within the posterior parietal cortex tend to be encoded in retinocentric coordinates, as demonstrated in event-related fMRI (Sereno et al., 1995; Medendorp et al., 2003; Merriam et al., 2003; Beurze et al., 2010), single-pulse TMS (van Donkelaar et al., 2000, 2002) and psychophysical patient studies (Khan et al., 2005a,b). Reach-related activity was found to depend on the retinotopic location of the target (Prado et al., 2005). Reaching to central targets activates the medial bank of the IPS bilaterally, whereas reaching to peripheral targets involves an additional region within the postero-superior part of the parieto-occipital sulcus. The latter activation is determined by the extra-foveal location of the target, which also entails poorer accuracy of the reaching movement.

## Functional MRI Studies of Prismatic Adaptation

The effect of prismatic adaptation has been investigated with PET (Clower et al., 1996) or with fMRI (Danckert et al., 2008; Luauté et al., 2009; Chapman et al., 2010; Crottaz-Herbette et al., 2014, 2017a,b,^2019^; Tissieres et al., 2018). Since fMRI yield better spatial resolution, they are likely to provide better insight into putative neural mechanisms. The relevant publications were recently summarized in a comprehensive review, which we recommend for further reading (Boukrina and Chen, 2021).

### Activation Patterns During Prism Exposure

**FIGURE 1:**
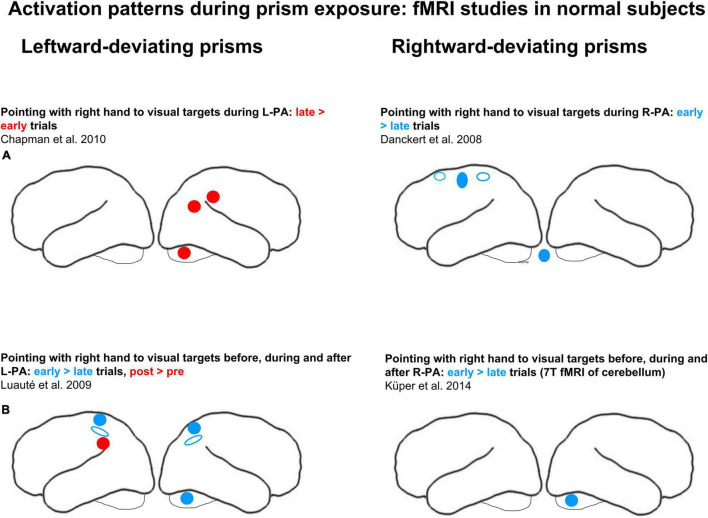
Effects of adaptation to left-deviating (left column) and right-deviating prisms (right column), as demonstrated in normal subjects with fMRI paradigms. **(A)** Changes of activity elicited by pointing during prism exposure. **(B)** Changes of activity elicited by pointing before, during, and after adaptation. Red and blue denote, respectively, increases and decreases of activity when comparing early vs. late stages of adaptation or pre- vs. post-adaptation sessions. Filled circles and ellipses mark cluster of activity on the convexity, empty ones those in sulci or on the medial part of the hemisphere.

Activation patterns during prism exposure have been investigated in *normal subjects* in four studies, two during R-PA and other two during L-PA (see schematic summary in Figure 1). In a first study event-related fMRI was carried out, while subjects were wearing right-deviating prisms and pointing with their right hand to visual targets (Danckert et al., 2008). It revealed clusters in the primary motor cortex, the anterior cingulate cortex and the anterior part of IPS on the left side and in the cerebellar vermis, where activity was higher during early than late trials. As the authors pointed out, this transient effect is likely to reflect the visuo-motor transformation induced by prisms. A second study of R-PA used 7T fMRI and limited the analysis to the cerebellum (Küper et al., 2014). Cerebellar lobules VIII and IX as well as the dentate nucleus were shown to participate in strategic motor control responses; greater activity during early than late trials was found in right lobule VIII.

Two studies investigated activation patterns during exposure to left-deviating prisms. In a first one, subjects pointed with their right hand to visual targets while they were wearing a left-deviating prism over the left eye (and a neutral lens over the right eye; Chapman et al., 2010). The analysis in this study was limited to regions of interest that were identified with a task of pointing to visual targets without prisms: right angular gyrus, right anterior IPL, right SPL, left SPL, left IPL as well as right and left cerebellum. When pointing with left-deviating prisms, greater activation was observed during the late than early trials in the angular gyrus, in the anterior IPL and in the cerebellum on the right side. In a second study subjects pointed with their right index to visual targets viewed through a left-deviating prism, placed over the right or the left eye, as randomly assigned across subjects (Luauté et al., 2009). Greater activation during the early than late trials was observed bilaterally within IPS and SPL as well as in cerebellar lobules IV and V on the right side, whereas greater activation during the late than early trials was present in left IPL (see schematic summary in Figure 1). The authors interpreted this as the implication of anterior IPS in error detection and of the parieto-occipital sulcus (POS) in error correction.

In summary, visuo-motor adaptation during prism exposure was shown to affect regions known to be involved in reaching, notably IPS (Danckert et al., 2008; Luauté et al., 2009). In their seminal review, Panico et al. (2020) proposed that this region, together with SPL and the upper part of IPL, is involved in recalibration, i.e., the correction of pointing errors early in prism exposure. Interestingly, the detection of the error introduced by a full prismatic shift and the ensuing realignment of motor behavior, as investigated in the above quoted activation studies (Danckert et al., 2008; Luauté et al., 2009), is not crucial for the induction of aftereffects. The aftereffects were demonstrated even when strategic recalibration was avoided by using multi-step prism adaptation (Michel et al., 2007; Panico et al., 2018). Furthermore, the role of right vs. left hand in prism adaptation is unclear. Up to now all studies used the right hand (Danckert et al., 2008; Luauté et al., 2009; Chapman et al., 2010; Küper et al., 2014).

### Changes in Cognitive Representations Induced by Right-Deviating Prisms

Two studies investigated the effect of R-PA *in normal subjects* on tasks that are known to involve the posterior parietal cortex and compared activation patterns that were elicited before vs. after the exposure to R-PA. A first study analyzed changes in activation patterns elicited by three different paradigms, namely (i) detection of visual targets presented in the left, right or central visual field; (ii) task of visuo-spatial short-term memory; and (iii) task of verbal short-term memory (Crottaz-Herbette et al., 2014). A brief exposure to R-PA was found to lead to an increase in activation by left, right and central targets in left IPL and to a decrease in activation by right and central targets in right IPL (see schematic summary in Figure 1). As pointed out by the authors, the representation of the ipsilateral visual field was enhanced in the left and decreased in the right IPL, which corresponds to a switch of the known hemispheric dominance from the right to the left (Crottaz-Herbette et al., 2014; Clarke and Crottaz-Herbette, 2016). No shift in hemispheric dominance was observed for either of the short-term memory tasks. A second study analyzed changes in activation patterns elicited by the detection of auditory targets presented in the left, right or central space and compared them to those elicited by visual targets at similar locations (Tissieres et al., 2018). A brief exposure to R-PA was found to lead to an increase in activation by auditory targets in left, right, and central locations in left IPL and to a decrease in activation by right auditory targets in right IPL (see schematic summary in Figure 1). Thus, the well known right hemispheric dominance for sound localization (Spierer et al., 2009) is reversed by a brief exposure to R-PA.

**FIGURE 2:**
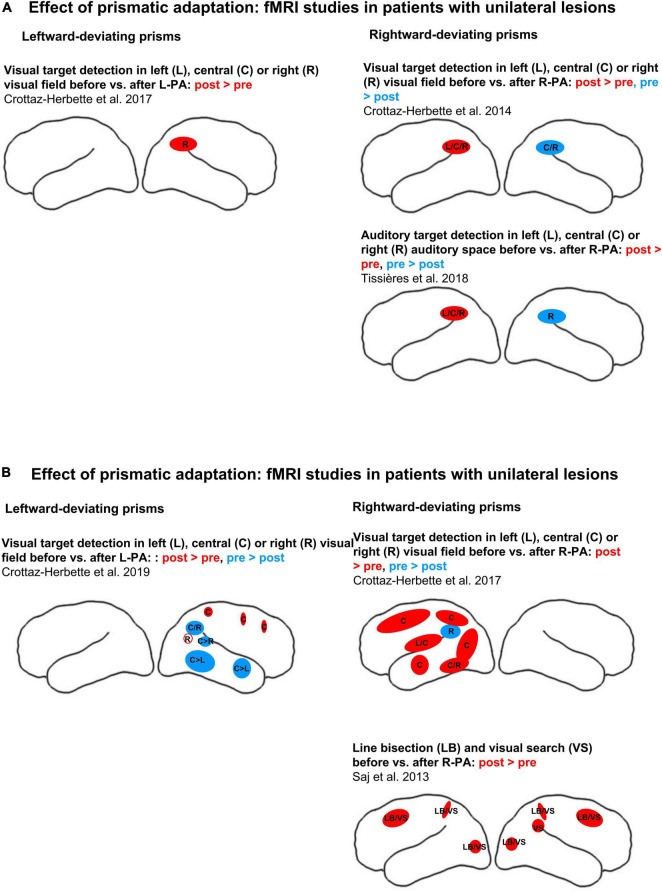
Effects of adaptation to left-deviating (left column) or right-deviating prisms (right column), as demonstrated with fMRI paradigms. **(A)** Changes of activity elicited by visual target detection before vs. after prismatic adaptation in normal subjects. **(B)** Changes of activity elicited by visual target detection (top row) or by line bisection and visual search before vs. after prismatic adaptation (bottom row) in patients with left or right unilateral hemispheric lesions (left and right columns, respectively). Red and blue denote, respectively, increases and decreases of activity when comparing early vs. late stages of adaptation or pre- vs. post-adaptation sessions. Filled circles and ellipses mark cluster of activity on the convexity, empty ones those in sulci or on the medial part of the hemisphere. L, C, and R denote, respectively, left, central, and right stimulus position in target detection paradigms. LB denotes line bisection, VS visual search.

Two other studies investigated *patients with right hemispheric lesions*, comparing activation patterns elicited by specific tasks before vs. after a brief exposure to R-PA (see schematic summary in Figure 2). In one study, a task known to involve VAN, detection of visual targets, was used (Crottaz-Herbette et al., 2017a). Within the left hemisphere it revealed an increase in activation by left targets within a region comprising the superior temporal gyrus, the insula and the lower part of the precentral gyrus. For central targets, increased activation was observed in the superior and middle temporal gyri, IPL, precuneus, middle and superior frontal gyri, posterior cingulate, and extrastriate occipital cortices. For right targets, increased activation was observed in middle occipital and inferior temporal gyri and decreased activation within the supramarginal gyrus. As pointed out by the authors, these results correspond to an enhancement of the representation of the ipsilateral and central visual field within the left hemisphere.

Line bisection and visual search were used as tasks in another study of patients with right-hemispheric lesions and neglect (Saj et al., 2013). In normal subjects the line bisection task was found to activate SPL and IPL bilaterally, with a predominance on the right side (Fink et al., 2001). Different aspects of visual search involve IPL and IPS, as demonstrated by activation studies for the efficiency of visual search (Nobre et al., 2003) or the degree of difficulty and the conjunction of features (Donner et al., 2002; Ischebeck et al., 2021). The right superior temporal gyrus was shown to contribute to particular form of visual search, the very difficult feature search (but not easy feature search or difficult conjunction search (Ellison et al., 2004; Gharabaghi et al., 2006; Ellison, 2008). In patients with right-hemispheric lesions and neglect, R-PA enhanced activity elicited by line bisection and by visual search within SPL, the superior frontal gyrus and the lateral occipital cortex on the left side (Saj et al., 2013). In the right hemisphere a similar increase of activity was observed within spared parts of the parietal, prefrontal and occipital cortex. Thus, R-PA restored task-related activity within regions, which are known to be involved in line bisection or visual search. The authors concluded that this bilateral activation reflects the recruitment of attentional networks, which leads to the alleviation of neglect symptoms.

### Changes in Cognitive Representations Induced by Left-Deviating Prisms

One study analyzed changes in activation patterns in normal subjects (Crottaz-Herbette et al., 2017b). Activation was elicited by a paradigm of detection of visual targets that were presented in the left, right or central visual field. A brief exposure to L-PA was found to lead to an increase in activation by right targets in right IPL. As pointed out by the authors, the increased ipsilateral visual field representation within the right IPL appears to reinforce the known right-hemispheric dominance within VAN.

One study was carried out with *patients with left hemispheric lesions*, comparing activation patterns elicited by visual target detection before vs. after L-PA (Crottaz-Herbette et al., 2019; schematic summary in Figure 2). It reported a wide-spread decrease in activation within the right temporo-occipital cortex and IPL; the decrease concerned mostly central and left stimuli and only to a very limited extent right stimuli. In addition, small clusters of activation increase were found in the right precuneus, elicited by right stimuli, and in right SPL and dorsal frontal convexity, elicited by central stimuli. It is noteworthy that in a subgroup of patients, who presented lateralized attentional deficits, L-PA enhanced the activation of the right IPL by ipsilateral, right stimuli (not represented in Figure 2). These results suggest that L-PA may reinforce right-hemispheric dominance within VAN in patients with right neglect (following left-hemispheric lesions), but in addition that it down-regulates the contralateral visual field representation within higher-order visual areas of the right hemisphere.

## Prism-Induced Topographical Mismatch Reshuffles Spatial Representations in Inferior Parietal Lobule

**FIGURE 3:**
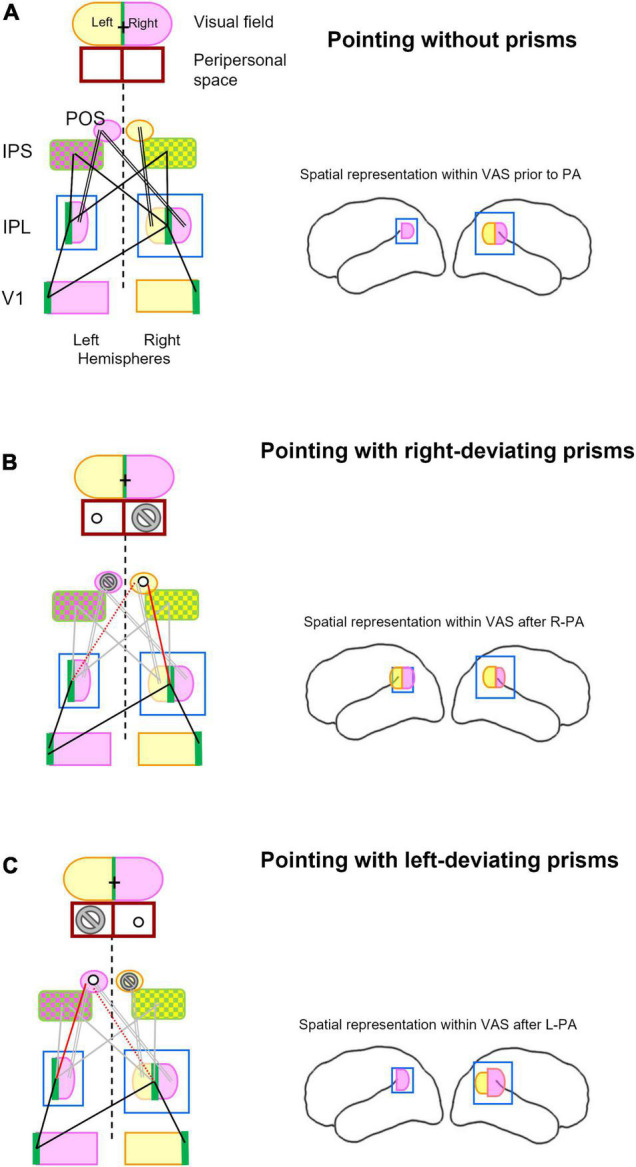
Schematic representation of key structures involved in visual pointing and their topographical relationship while pointing without prism **(A)**, with right-deviating prisms **(B)**, and with left-deviating prisms **(C)**. Lateral views of the left and right hemispheres in right-hand column summarize spatial representation within the inferior parietal lobule and its changes after prismatic adaptation as described previously (Crottaz-Herbette et al., 2014, 2017b; Tissieres et al., 2018). **(A)** The visual field is subdivided into right (violet), left (yellow), and vertical meridian parts (green). Left and right halves of peripersonal space are outlined below the visual field figurine and are aligned with it, representing thus the situation of pointing to central (visual) targets. The same color code, violet for right, yellow for left, and green for central visual field, is used for the respective representations within the primary visual area (V1), the inferior parietal lobule (IPL), the intraparietal sulcus (IPS), and the parieto-occipital sulcus (POS). For simplicity reason, the extra-striate visual areas are not included. Whereas IPS encodes central (green) and contralateral targets (violet and yellow, respectively), POS encodes only the contralateral targets. Pointing to visual targets at (central) fixation is the most accurate (Prado et al., 2005) and most commonly adopted. Without prisms, pointing to central target involved central visual field representations (green) in visual areas (here represented by V1), IPL and IPS. Main topographic relationships involved in pointing to central targets are indicated here by single black lines, those involved in pointing to peripheral targets by double black lines. Brain figurine on the right depicts bilateral space representation in right IPL. **(B)** Pointing to visual targets while wearing right-deviating prisms implies creating a relationship between the visually perceived target at fixation point (cross in visual field; green in V1 and IPL) and the actual target in the peripersonal space to the left of the fixation point (circle). It is to be noted that the actual target will never be in the peripersonal space to the right of the fixation point (marked by the no-go sign). This new configuration weakens links between the central representations within IPL and IPS on either side (gray lines) and between right space representation within right and left IPL and the left IPS and POS (double lines in gray). It will establish a new link between the central representation in IPL on the right (and possibly the left) side and the left space representation within right IPS and POS (red line), possibly by re-adjusting a previous link between the left space in these structures. Brain figurine on the right depicts R-PA-induced bilateral space representation in left IPL (Crottaz-Herbette et al., 2014; Tissieres et al., 2018). **(C)** Pointing to visual targets while wearing left-deviating prisms implies creating a relationship between the visually perceived target at fixation point (cross in visual field; green in V1 and IPL) and the actual target in the peripersonal space to the right of the fixation point (circle). It is to be noted that the actual target will never be in the peripersonal space to the left of the fixation point (marked by the no-go sign). This new configuration weakens links between the central representations within IPL and IPS on either side (gray lines) and between left space representation within right IPL and right IPS and POS (double line in gray). It will establish a new link between the central representation in IPL on the left (and possibly right) side and the right space representation within the left IPS and POS (red line), possibly by re-adjusting a previous link between the right space in these structures (dotted red line). Brain figurine on the right depicts L-PA-induced reinforcement of right space representation in right IPL (Crottaz-Herbette et al., 2017b).

We propose here a model (Figure 3), which offers a parsimonious explanation of the effect of R-PA and L-PA. This model posits that the prism-induced topographical mismatch in the posterior parietal cortex reshuffles spatial representations within IPL, which then impacts on attentional and visuo-spatial functions. The term “reshuffle” describes here rapidly occurring change in spatial representations, which is due to the weakening and readjusting of input–output relationship. The reshuffled spatial representations can contribute to attentional and visuo-spatial processing but with a relative loss of efficiency. The key feature of this model, namely the enhancement of the ipsilateral space representation on the left side following R-PA and on the right side following L-PA has been reported in prior studies (Crottaz-Herbette et al., 2014, 2017b; Tissieres et al., 2017).

The mechanisms that lead to the reshuffling of spatial representations in IPL can be understood in terms of topographic relationships in the posterior parietal cortex. The representation of central and contralateral visual field within the primary (V1) and within extrastriate visual areas (Tootell et al., 1998) in either hemispheres has strong functional interactions (Ruff et al., 2008) with the right-dominant IPL (Thiel et al., 2004; Ruff et al., 2008; Beume et al., 2015). The underlying pattern of neural connections has been partially demonstrated in post-mortem anterograde tracing studies: retinotopically organized connections between early-stage visual areas (Clarke, 1994) as well as homo- and heterotopic callosal connections (Clarke and Miklossy, 1990; Di Virgilio and Clarke, 1997).

Pointing to a visual target involves parts of the posterior parietal cortex that encode reach-related space (Sereno et al., 1995; van Donkelaar et al., 2000; Medendorp et al., 2003; Merriam et al., 2003; Beurze et al., 2010). The visual input to the posterior parietal cortex is believed to be mediated by a cascade of cortico–cortical connections. Direct, monosynaptic intra- and interhemispheric connections were demonstrated in post-mortem tracing studies between visual areas (Clarke and Miklossy, 1990; Clarke, 1994) as well as from extrastriate visual areas to the posterior parietal cortex (Di Virgilio and Clarke, 1997). Structural white matter pathways were identified by *in vivo* tractography, linking regions of the parieto-temporal junction to SPL and IPS (Makris et al., 2013, 2017; Kamali et al., 2014; Wu et al., 2016). These connections provide the anatomical basis for *functional links*, which are effective in pointing. Whereas reaching to central targets involves the medial bank of the IPS bilaterally (IPS in Figure 3), reaching to peripheral targets relies on an additional region within the postero-superior part of the contralateral POS (Prado et al., 2005). As outlined in Figure 3A, there is a *functional link* between the representations of central and peripheral visual field in IPS and POS and in IPL.

The most accurate (Prado et al., 2005) and most commonly adopted pointing to visual targets makes use of central fixation. This is also generally the case when subjects point to targets while wearing prisms. Wearing *right-deviating prisms* creates a situation, where the actual target is always left of the fixation point (Figure 3B). This changes the topographic relationship between right and left IPL and the reach area in IPS and POS. More specifically, central space representation in right IPL becomes linked to left space representation in right IPS and POS. Since reaching into the right space does not occur in this particular situation, the links between IPS & POS and IPL in the left hemisphere are likely to weaken. This temporary loss of functional relationship may favor the appearance of ipsilateral spatial representation within left IPL, as demonstrated in previous studies (Crottaz-Herbette et al., 2014; Clarke and Crottaz-Herbette, 2016; Tissieres et al., 2018). This R-PA-induced change in responsiveness within left IPL reflects very likely its prior functional characteristics, such as its contribution to visuo-spatial attention in the specific Posner task condition of expectancy (Doricchi et al., 2010). Alternatively, after the exposure to R-PA visual stimuli may gain access to the left-dominant motor attentional network (Rushworth et al., 2001, 2003) or the left-dominant implicit representation of the auditory space (Tissieres et al., 2019). This mechanism would explain the R-PA-induced switch in dominance of VAN from the right to the left hemisphere, reported for visual and auditory stimuli in normal subjects (Crottaz-Herbette et al., 2014; Tissieres et al., 2018).

Pointing to a central target while wearing *left-deviating prisms* creates a situation, where the actual target is always right of the fixation point (Figure 3C). This changes the topographic relationship between right and left IPL and the reach area in IPS and POS. More specifically, central space representation in left IPL becomes linked to right space representation in left IPS and POS. Since reaching into the left space does not occur in this particular situation, the links between IPS & POS and IPL in the right hemisphere are likely to weaken. These changes may introduce fuzziness in spatial representation in the right IPL and lead to the enhancement of ipsilateral visual field representation within the right IPL, as described in a previous study (Crottaz-Herbette et al., 2017b). The enhanced right space representation in right IPL may lead to greater activity with the right space representation within IPS and SPL on the left side. This is compatible with the previously reported change in excitability of the parietal circuitry, with an increase on the left and decrease on the right side (Schintu et al., 2016; Martín-Arévalo et al., 2018).

**FIGURE 4:**
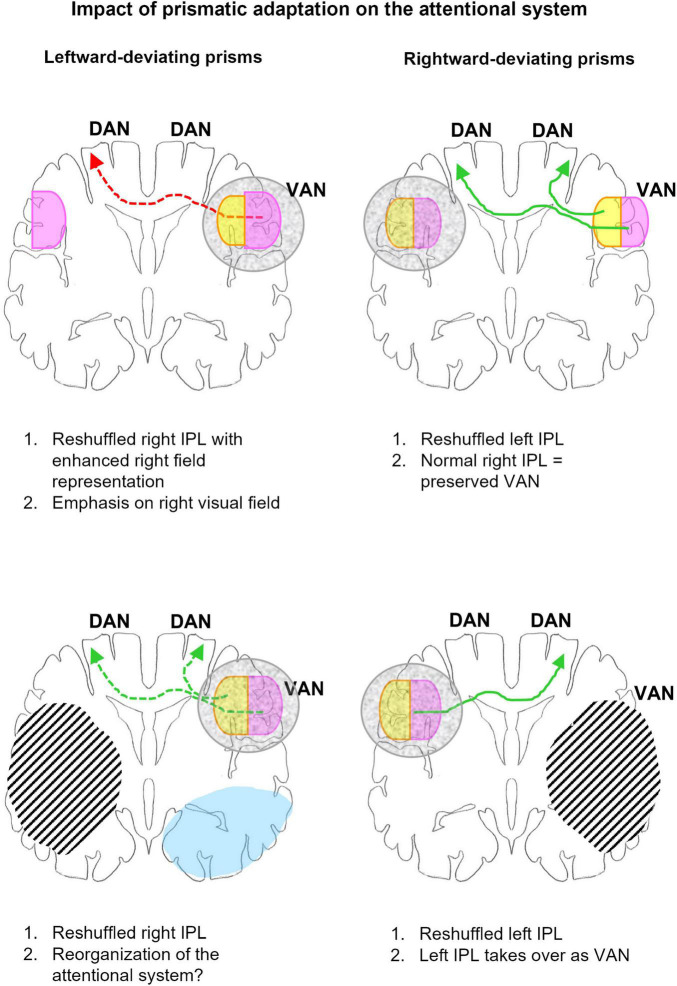
Schematic representation of the impact of adaptation to left- (left column) and right-deviating prisms (right column) on the organization of the attentional network in normal subjects (top row) and patients with unilateral hemispheric lesions (bottom row). The outline represents a coronal section at the level of IPL, right hemisphere is to the right. Gray mottled overlay marks reshuffled IPL representations. Right space representation is in violet, left space in yellow. Green arrows indicate links relevant to correct or recovered function, red arrows those contributing to dysfunction. Arrows in full line mark links compatible with behavioral and/or imaging evidence, those in dashed line, hypotheses to be tested. Hatching represents lesions, blue, regions where L-PA downregulates activation by visual stimuli. *Top*: In normal subjects L-PA leads to the reshuffling of spatial representations within VAN, enhancing the ipsilateral space representation (Crottaz-Herbette et al., 2017b). The latter is likely to increase the activation of DAN in the left hemisphere. Both the relative dysfunction of the reshuffled VAN and increased activation of left DAN are likely to contribute to the neglect-like effects, which L-PA induces in normal subjects. R-PA leads in normal subjects to the reshuffling of spatial representations within left IPL, by enhancing the ipsilateral space representation (Crottaz-Herbette et al., 2014; Tissieres et al., 2018). Since VAN is preserved, the reshuffled left IPL is very likely not to impact attentional functions. *Bottom*: In patients with right-hemispheric lesions, R-PA leads to the reshuffling of spatial representations within left IPL, by enhancing the ipsilateral space representation (Crottaz-Herbette et al., 2017a). In the absence of a functional VAN, the left IPL takes over and drives DAN on either side. In patients with left-hemispheric lesions, L-PA reshuffles and changes partially spatial representations within VAN; in addition it downregulates visual activity in the extrastriate cortex (Crottaz-Herbette et al., 2019). The reorganization of VAN may have a positive impact on attentional functioning.

In summary, the use of right- or left-deviating prisms leads to the reshuffling of spatial representations within IPL, which predominates contralateral to the direction of prism deviation (Figure 4). The presumed mechanisms are similar for either direction of deviation. Successful pointing to central targets while wearing right-deviating prisms involves reaching always to the left of the fixation point, never to the right. The under-use of the right space downregulates the activity within the reach-relevant IPS & POS and IPL on the left side and results in the reshuffling of spatial representations in left IPL. The symmetrical effect is observed with left-deviating prisms. The under-use of the left space downregulates the activity within the reach-relevant IPS & POS and IPL on the right side and results in the reshuffling of spatial representations in right IPL. It is very likely that reshuffled spatial representations in IPL have some functional competence, i.e., they can participate in attentional and visuo-spatial functions. However, it is reasonable to presume that they are less efficient than regular representations and may thus cause partial dysfunction.

### PA-Induced Changes in Functional Connectivity

Supporting evidence for PA-induced reshuffling of the attentional network comes from studies of functional connectivity. Four studies have compared resting-state connectivity before vs. after an exposure to PA. Two of them have contrasted the effect of right-deviating prisms and that of plain glasses. In a first study, global connectivity analysis revealed significant decreases in functional connectivity of nodes within left IPL and left insula as well as bilaterally within the medial prefrontal cortex, revealing a reduction of the connectivity between the Default Mode Network and the attentional network (Wilf et al., 2019). In a second study, whole brain analysis using the graph theory approach revealed a decrease in connectivity strength and in local efficiency in VAN, but not DAN (Gudmundsson et al., 2020). A third study carried out seed-based correlation analysis within the attentional network before and after a brief exposure to R-PA, without a control group (Tsujimoto et al., 2019). This comparison showed changes in connectivity within the right hemisphere, a decrease between IPS and the frontal eye field and an increase between the frontal eye field and the anterior cingulate cortex. A fourth, elegant study contrasted the effect of R-PA vs. L-PA using seed-based analysis and showed a decrease in resting state connectivity in the spatial navigation network, i.e., right posterior parietal cortex, hippocampus, and cerebellum (Schintu et al., 2020b). More specifically, R-PA increased and L-PA decreased connectivity within parts the posterior parietal cortex and from it to the right middle frontal gyrus.

Thus, PA-induced reorganization involves indeed changes in functional links, as postulated by our model. However, current studies, both with seed-based or whole-brain approaches, do not have the necessary spatial and temporal resolution to investigate the fine-tuning of visuo-motor recalibration or its impact on visuo-spatial and other cognitive representations.

### Putative Mechanisms of Spatial Reshuffling

The reshuffling of spatial representations in IPL occurs very rapidly, after an exposure of barely few minutes to R-PA (Crottaz-Herbette et al., 2014, 2017a; Tissieres et al., 2017) or L-PA (Crottaz-Herbette et al., 2017b,^2019^). Such a short time frame speaks in favor of PA—induced instability of the synaptic organization and a rapid emergence of pre-existing spatial representations. Axonal sprouting and/or synaptic remodeling, which would require days or weeks (Nudo, 2006), are thus ruled out for this stage of PA.

The posterior parietal cortex is part of the dorsal visual stream (Ungerleider and Mishkin, 1982). It subserves different aspects of spatial vision, including location of objects and grasping but also different aspects of motion analysis (Galletti and Fattori, 2018). Different visual sub-streams have been identified within the occipito-parietal cortex of non-human primates (e.g., Galletti et al., 2003; Rizzolatti and Matelli, 2003). As summarized in a recent review (Galletti and Fattori, 2018), the different networks appear to be dynamically activated or inhibited according to context. They do not constitute a fixed ensemble of cortical areas, which are involved only in one specific function, but interconnected neural networks, where the same neurons participate in several functional processes and whose activation changes according to the context. It is very likely that the human posterior parietal cortex is organized in a similar fashion, with multiple interconnected networks, whose activity is modulated by the context. As posited by our model, a brief exposure to prisms creates an instability in the visuo-motor network, which changes the configuration of neural networks within VAN and results in the emergence of new ipsilateral spatial representations in IPL.

Very little is known about the duration of PA-induced changes in IPL spatial representations. In normal subjects, they may subside with the disappearance of cognitive after-effects, i.e., after a few hours (e.g., Crottaz-Herbette et al., 2014), but there is currently no imaging evidence to this effect, neither in normal subjects nor in patients with unilateral lesions. Maintaining PA-induced spatial representations in IPL is of interest in patients with neglect. It is currently unknown whether a daily exposure to R-PA combined with other rehabilitation interventions over several weeks (e.g., Frassinetti et al., 2002) leads to a stabilization of left IPL spatial representations. Recent, highly interesting studies open a promising line of investigation into the prolongation of PA effects; they demonstrated that stimulation of the primary motor cortex by tDCS strengthens PA-induced aftereffects and boosts the therapeutic effect of R-PA in neglect (review: Panico et al., 2021).

### Cerebellar Contribution to Prismatic Adaptation

As pointed out in a recent review, the cerebellar-parietal network is crucial for cognitive aftereffects to occur (Panico et al., 2018). The contribution of the two key structures has been highlighted in patients with focal brain damage. Whereas bilateral lesions of the posterior parietal cortex, centered on SPL, were shown not to interfere with visuo-motor prism adaptation (to left-deviating prisms; Pisella et al., 2004), they appear to preclude the reduction of rightward attentional bias (following R-PA; Striemer et al., 2008).

Cerebellar dysfunction was shown to impair prismatic adaptation and in particular to decrease the visuo-motor aftereffect, both in patients (Weiner et al., 1983; Martin et al., 1996) and in non-human primates (Baizer et al., 1999. The effect is side related, as demonstrated in a patient, who suffered stroke in the territory of the left superior cerebellar artery and who adapted to rightward but not to leftward prism, independently of the hand used during exposure (Pisella et al., 2005). As outlined in an influential review, the cerebellar hemisphere ipsilateral to the prismatic displacement appears to be crucial for prism adaptation (Pisella et al., 2006). Lesion of the left cerebellar hemisphere, known to be interconnected with the right cerebral cortex, interfered most likely with the reorganization of the right parietal cortex induced by L-PA, but not with that of the left parietal cortex induced by R-PA. Thus, the absence of adaptation to leftward prisms following a left cerebellar lesion, as reported by Pisella et al. (2005), is compatible with our model; the left cerebellar lesion prevented the L-PA-induced re-organization within the right parietal cortex.

The importance of the contribution of the cerebellar hemisphere ipsilateral to the direction of prism deviation and of neural reorganization within the parietal cortex contralateral to it was addressed in three elegant tDCS studies in normal subjects. The aim of these studies was to disentangle the role of the cerebellum during the recalibration vs. realignment phases of R-PA. In all three studies continuous cathodal tDCS was applied over the right cerebellum and pointing was performed with the right hand. In a first study, tDCS started before R-PA and continued during adaptation and aftereffect testing; it led to greater rightward deviation during the first trials with prisms and to a greater leftward deviation during the aftereffect, indicating that both phases were affected (Panico et al., 2016). In a second study, PA was administered in a multistep fashion with progressively increasing prism deviations and tDCS was applied only during prism exposure; it led to a greater rightward deviation during the initial, but not the subsequent trials of each deviation step, demonstrating cerebellar involvement in spatial realignment (Panico et al., 2018). In a third study, anodal or cathodal tDCS was applied simultaneously over the left parietal cortex and the right cerebellum during the whole PA procedure. The comparison of the two conditions showed reduction of terminal errors during exposure to prims under anodal and reduction of the aftereffect under cathodal tDCS, indicating the contribution of the parieto-cerebellar network both to recalibration and realignment processes (Panico et al., 2022). These three studies highlighted the contribution of the right cerebellum and of the left parietal cortex to R-PA. Their results are compatible with our model, which posits critical reorganization within the left parietal cortex during R-PA.

### Direction of Prims Deviation vs. Hand Used During Adaptation

There is partial evidence that the reshuffling of IPL contralateral to prism deviation depends indeed on the direction of prism deviation and not on the hand used. A new study compares the effect of R-PA executed with the right hand with that of two control conditions, namely (i) R-PA executed with the left hand, and (ii) L-PA executed with the right hand (Farron et al., 2022 in revision). This study confirmed the previously described enhancement of the representation of left central space within left IPL. The use of right vs. left hand during adaptation modulated this enhancement in some, but not all parts of left IPL. The use of right hand with L-PA mimicked partially the effect by enhancing the response to ipsilateral stimuli in left IPL and decreasing it in right IPL.

### Adaptation to Prismatic Deviation by Eye Movements

Several studies described a procedure for prismatic adaptation by means of repeated gaze shifts toward targets, and not hand movements (Michel et al., 2013; Ronga et al., 2017a,b; Saj et al., 2019). With left-deviating prisms, this procedure yielded in normal subjects rightward bias in visual straight ahead and line bisection tasks. With right-deviating prisms, this procedure yielded visuo-motor aftereffects in normal subjects (Michel et al., 2013) and a decrease of neglect severity in straight-ahead and paper-and-pencil tasks in patients (Ronga et al., 2017a). This procedure with right-deviating prisms enhanced neural activation elicited by bisection and by visual search tasks in patients with right frontal but not parietal lesions (Saj et al., 2019). It is currently unclear, whether prismatic adaptation by means of repeated gaze shifts relies on the same neural mechanisms as prismatic adaptation by means of hand movements. Within the posterior parietal cortex, there are regions selective for reaching and those involved in both reaching and saccades. The parietal reach region responds preferentially during reaching (rather than during saccades; Connolly et al., 2003). Other parts of the posterior parietal cortex support both reaching and saccades toward a visual target (Beurze et al., 2009). It is reasonable to expect that oculomotor prismatic training (Ronga et al., 2017a) may affect VAN in the same way as does PA. This needs, however, to be established in activation studies, such as those using the target detection paradigms (Crottaz-Herbette et al., 2014, 2017b).

## The Side of Inferior Parietal Lobule Reshuffling Determines Behavioral Effects of Prismatic Adaptation

The side-specific reshuffling of spatial representations within IPL can account for the absence of behavioral consequences after R-PA in normal subjects, for their presence after L-PA, as well as for the alleviation of neglect symptoms with R-PA in patients with right-hemispheric lesions. Furthermore, it offers a hypothesis for new investigations of the effect of L-PA in patients with left-hemispheric lesions.

### Rightward Prismatic Adaptation

In the case of *R-PA*, the striking aspect is the paucity of behavioral effects *in normal subjects*. A series of studies reported absence of significant visuo-spatial or cognitive effects of R-PA in normal subjects (Loftus et al., 2009; Bultitude and Woods, 2010; Goedert et al., 2010; Fortis et al., 2011; Schintu et al., 2014, 2017; Michel et al., 2019), whereas two reported very specific ones. Using the Posner paradigm, Striemer et al. (2006) found that R-PA speeded up reflexive reorienting from invalid cues on the left to targets on the right side in subjects with initially large cueing effects. A second study reported rightward shift in visual midpoint judgment in extrapersonal space (but not in peripersonal space; Berberovic and Mattingley, 2003).

The paucity of behavioral effects of R-PA in normal subjects is very likely due to the fact that R-PA reshuffles IPL on the non-dominant, left side, and that the right-dominant VAN maintains normal function. However, this hypothesis does not offer a parsimonious explanation for the very specific effects on invalid cues in the Posner paradigm (Striemer et al., 2006) or on midpoint judgment in the extrapersonal space (Berberovic and Mattingley, 2003). It is to be noted, that a population of healthy, right-handed subjects may be heterogeneous in respect to eye dominance. A recent study demonstrated that the normally occurring leftward bias tends to be smaller in subjects with left than right eye dominance (Schintu et al., 2020a). These results highlight the necessity for future studies on PA not only to take into account hand but also eye preference of the subjects.

The absence of significant visuo-spatial and cognitive effects of R-PA in normal subjects contrasts with the alleviation of neglect symptoms in *patients with right-hemispheric lesions* reported for the first time by Rossetti et al. (1998). Numerous studies followed reporting improvement in spatial cognition as well as in activities of daily living, as summarized in seminal reviews (Pisella et al., 2006; Barrett et al., 2012; Newport and Schenk, 2012; Jacquin-Courtois et al., 2013; Champod et al., 2018; De Wit et al., 2018; Barrett and Houston, 2019). The numerous reports of beneficial effects of R-PA on neglect symptoms in individual patients or in small groups of patients (above) contrast with the negative results in terms of rehabilitation outcome in large scale clinical trials (e.g., Ten Brink et al., 2017; Qiu et al., 2021; Scheffels et al., 2021).

The beneficial effect of R-PA on neglect symptoms, as reported in numerous studies (Pisella et al., 2005; Barrett et al., 2012; Newport and Schenk, 2012; Jacquin-Courtois et al., 2013; Champod et al., 2018; De Wit et al., 2018; Barrett and Houston, 2019), can be explained by the reshuffling of left IPL, which is accompanied by the emergence of ipsilateral space representation. We propose that the reshuffling of spatial representations within the left IPL renders the left-dominant attentional network (Rushworth et al., 2001, 2003) and the left-dominant implicit representation of the auditory space (Tissieres et al., 2019) accessible to visual stimuli, creating thus an alternative visual space representation, which takes over the role of the damaged right-dominant VAN. The ensuing switch of dominance within VAN from the right to the left hemisphere has been described and commented upon in previous publications (Crottaz-Herbette et al., 2014, 2017a; Clarke et al., 2015; Clarke and Crottaz-Herbette, 2016; Tissieres et al., 2018).

The lack of positive results of R-PA on rehabilitation outcome, which has been reported in large scale clinical trials on neglect (e.g., Ten Brink et al., 2017; Qiu et al., 2021; Scheffels et al., 2021), indicates that some, but not all patients with neglect respond equally well to R-PA. These findings are entirely compatible with our model and have been indeed predicted in previous publications. In particular, it has been postulated that the beneficial effect of R-PA on neglect symptoms requires a preserved link between the left IPL to DAN on either side (Clarke et al., 2015; Clarke and Crottaz-Herbette, 2016). This latter point has been confirmed by reports of responders to R-PA, who tend to have preserved posterior callosal pathway and preserved right SPL (Tissieres et al., 2017; Goedert et al., 2020; Gutierrez-Herrera et al., 2020). Furthermore, neglect patients with frontal lesions not only showed larger benefits of R-PA than patients with parietal lesions but tended also to recruit larger parts of right parietal areas when executing line bisection and visual search tasks (Saj et al., 2019).

In summary, R-PA leads to reshuffling of left IPL with the emergence of an ipsilateral space representation. The reshuffling of the left IPL is beneficial in neglect, since it provides an alternative to the damaged right VAN. Normal subjects continue to rely on VAN in their right hemisphere and do not take advantage of the reshuffled, less efficient left IPL.

### Leftward Prismatic Adaptation

Leftward prismatic adaptation was repeatedly reported to induce *in normal subjects* neglect-like performance in several visuo-spatial tests (Michel, 2016). We propose that these effects are the result of the reshuffling of the highly specialized right IPL, which becomes temporarily less efficient and mimics thus right-hemispheric dysfunction. Similarly to neglect, right IPL dysfunction is likely to entail hypoactivity in right DAN, which lowers its inhibition of left DAN and results hyperattention to the right space. In addition, as shown in a previous study, L-PA emphasizes the responsiveness of right IPL to right visual stimuli (Crottaz-Herbette et al., 2017b). This enhancement may facilitate the access of right stimuli to the left DAN and drive it more forcefully, leading to a right attentional bias in behavioral tasks.

Martín-Arévalo et al. (2016) investigated the effect of L-PA on the Posner task using event-related potentials (ERPs). They reported greater decrease of ERPs in response to left, as compared to right cues. Since these ERPs reflect neural activity within the IPS in relation with attentional orienting, this finding was interpreted as orienting bias toward rightward cues following L-PA. In addition, the authors reported smaller ERPs for the invalidly cued left than right targets. These results were interpreted as a deficit in the disengagement from the right space. The authors highlighted thus bilateral modulations of the DAN, the role of the interhemispheric connections, and interaction with the cerebellum. Their results and interpretation of the bilateral modulation of DAN are aligned with our previous findings (Crottaz-Herbette et al., 2014, 2017a,b,^2019^; Tissieres et al., 2018) and with our new model.

One of the best documented effects of L-PA is the induction of a rightward bias on the perceptual variant of the line bisection task (e.g., Colent et al., 2000; Michel et al., 2003; Michel and Cruz, 2015; Striemer et al., 2016; McIntosh et al., 2019), both in peri- and extrapersonal space following L-PA (Berberovic and Mattingley, 2003). This effect can be explained by a dysfunction of right IPL, which impacts on ipsi- and contralateral SPL, structures known to be involved in line bisection (Fink et al., 2001). Interestingly, L-PA effect on line bisection is long-lasting but fluctuating, which has been interpreted in terms of an underlying reorganization (Schintu et al., 2014). We propose that a reshuffling of right IPL is the core of this reorganization, leading to right IPL dysfunction and lesser right SPL activity, with ensuing hyperactivity within the left SPL, which results in neglect-like symptoms.

The temporary dysfunction of right IPL, as described in our model, offers also an explanation for the reported modulation of global vs. local processing bias induced by L-PA. Briefly, L-PA was shown to reduce the normally occurring global processing bias (Bultitude and Woods, 2010) and to enhance local processing bias (Reed and Dassonville, 2014). Imaging studies have shown that attending to global vs. local features implicates partially distinct neural networks (Fink et al., 1996, 1997). Directing attention to local aspects involves specifically the left inferior occipital cortex, whereas attending to global aspects the right lingual gyrus. The latter is likely to be disturbed by the reshuffling of spatial representation within the right IPL, which then results in the disadvantage of global feature processing.

The temporary dysfunction of right IPL, with the ensuing decrease of attention to the left, offers also an explanation for the reported effect of L-PA on the grayscales task. The naturally occurring bias to choose the darker card on the basis of the shade of gray on its left side is reversed after L-PA (Loftus et al., 2009). The same mechanism may be put forward for effects on cognitive representations, such as that of auditory frequencies along a horizontal axis (Michel et al., 2019). It is to be noted, however, that the decrease of attention to the left, i.e., the reduction of the pseudoneglect bias (Loftus et al., 2009) on gray scales could also be explained by inhibition of the right parietal cortex by the left cerebellum (Pisella et al., 2006).

Intriguing results were reported concerning the effect of L-PA on the detection of near-threshold stimuli (Ronga et al., 2018). The exposure to L-PA, but not a control condition without prisms, increased the percentage of perceived near-threshold gray rectangles, which were presented tachistoscopically; the effect was significant for stimuli presented both right and left of the fixation point. In addition, the response times were lower for stimuli on either side. The same study reported a second experiment, where the effect of R-PA on near-threshold detection was analyzed on its own (i.e., without a control group); the authors report increase of the percentage of perceived stimuli and decrease in response times. Near-threshold stimuli reported as seen were associated with greater activity within right and left IPL and with tighter coupling within a fronto-parietal network, highlighting the interaction between spatial attention and conscious perception (Chica et al., 2013). More specifically, there is evidence for interaction between phasic alerting and conscious perception of tachistoscopically presented near-threshold visual stimuli. Behavioral studies have shown that phasic alerting by auditory stimuli improves conscious perception (Kusnir et al., 2011). Activation studies have demonstrated that conscious perception of near-threshold stimuli relies on a network known to be involved in phasic alerting, namely the anterior cingulate cortex, supplementary motor area, frontal eye fields, thalamus, and caudate nucleus (Chica et al., 2016). As pointed out in a recent study, this attentional network may, however, be modulated not only by subjective visibility of the stimulus, but also by decision confidence (Mazor et al., 2022). The intriguing effect of L-PA on the detection of tachistoscopically presented near-threshold stimuli may thus reflect (i) greater impact of the known advantage of the left hemisphere in detecting transient events (Karim and Kojima, 2010); (ii) greater activation of the phasic alerting network (Chica et al., 2016); or a hitherto unknown modulation of the decision process (Mazor et al., 2022). Further studies are needed to investigate these three options.

The effect of *L-PA in patients with left hemispheric lesions* have not been investigated systematically. However, a recent study demonstrated a wide-spread reshaping of visuo-spatial representations within the intact right hemisphere (Crottaz-Herbette et al., 2019). Overall L-PA yielded a decrease of neural activity elicited by central and left visual targets within the temporal cortex and by right and central targets in IPL. In patients with lateralized attentional deficits, L-PA tended to emphasize right spatial representation in IPL. Further studies need to determine the long-term effects of such reshaping, with particular focus on lateralized vs. non-lateralized attentional deficits.

## Conclusion

Our model conveys a relatively simple message. Opting for R- vs. L-PA means choosing the side of major IPL reshuffling. The reshuffling predominates in IPL contralateral to the direction of prism deviation and is likely to lead to its partial dysfunction in normal subjects. In patients with right hemispheric lesions, R-PA-induced reshuffling leads to the recruitment of alternative spatial representations within left IPL, whereas in patients with left hemispheric lesions, L-PA-induced reshuffling leads to the recruitment of alternative spatial representations within right IPL.

Models of mechanisms underlying the effects of prismatic adaptation are not only of conceptual importance but they are also clinically relevant. Conceptually our model offers new hypotheses for experimental work. The following predictions derived from our model could be addressed in fMRI studies:

i) Normal subjects, which underwent R-PA, continue to use right IPL in attentional tasks (such as the Posner paradigm), whereas patients with neglect make use of the new spatial representation in left IPL.
ii) Normal subjects, who underwent L-PA, show activation patterns elicited by visuo-spatial tasks (such as tonic and phasic alertness, line bisection, and visual search) that are compatible with right IPL dysfunction.

Both these predictions are compatible with the electrophysiological markers recorded in normal subjects during the Posner paradigm (Martín-Arévalo et al., 2016). In particular, this study has shown that L-PA yields asymmetries in attentional orienting and in attentional disengagement from invalidly cued targets reminiscent of neglect and indicating hence putative dysfunction of right VAN. The absence of electrophysiological effects following R-PA, described in this study, suggests that right IPL continues to be used. The outlined fMRI studies would lead to a precise identification of the regions involved.

In clinical practice, the understanding of the neural mechanisms underlying R-PA are essential to refine indications for this treatment in unilateral neglect and to focus future clinical trials (Clarke et al., 2015). For L-PA, our model may lead to new approaches when treating attentional deficits in cases of left hemispheric lesions.

## Author Contributions

All authors listed have made a substantial, direct, and intellectual contribution to the work, and approved it for publication.

## Conflict of Interest

The authors declare that the research was conducted in the absence of any commercial or financial relationships that could be construed as a potential conflict of interest.

## Publisher’s Note

All claims expressed in this article are solely those of the authors and do not necessarily represent those of their affiliated organizations, or those of the publisher, the editors and the reviewers. Any product that may be evaluated in this article, or claim that may be made by its manufacturer, is not guaranteed or endorsed by the publisher.
